# Four Children at a Birth, Averaging Five Pounds Each

**Published:** 1867-08

**Authors:** C. S. Faust

**Affiliations:** Graham’s Turn-Out, South Carolina


*Four Children at a Birth, averaging five pounds each; all living and well, and nursing the mother.* By C. S. Faust, M. D., Graham's Turn-Out, South Carolina.

Some few weeks ago, the following letter was received from a graduate of the New York University, dated Graham's Turn-Out, March 7, requesting any remarks which I might think proper to make, in reference to the interesting case which he details. Accordingly, after reading the case, I thought it of sufficient importance to the readers of your journal to give it in full, as presented to Dr. Faust. I have also taken the liberty of adding my comments on the same.

I am, sir, very truly,
Gunning S. Bedford, M. D.

Graham's Turn-Out, S. C.,
March 7, 1867.

Prof. G. F. Bedford, 66 Fifth Avenue, New York:

*My Dear Doctor*,—I have just visited a lady who has been delivered of four healthy mail children at one birth. One of the children was born on the 26th of last February, at 11 o'clock, a. m., the other three were born on the following day (27th,) between the hours of 6 and 8 o'clock, a. m. There was but one placenta, which was square; cord attached to each corner. The mother is just twenty-five years of age, and is doing well. All the children nurse the mother, and will average five pounds each in weight.

Thinking, perhaps, that you had not seen such a case recently, and especially where all the children were living and doing well, I deemed it my duty, as an old student of yours, to inform you of the fact, as obstetrics was always your favorite branch. Please let me know what you think of such cases, and how often they occur.

I remain yours, very truly,
C. J. Faust, M. D.

REPLY.

66 Fifth Avenue, New York,
March 20th, 1876.

*My Dear Doctor*—Your very kind letter of the 7th instant

I received with much interest. The case of quadruple birth which you described is, indeed, a remarkable one. You ask me "what I think of such cases, and how often they occur?" Well, I tell you very frankly, that *four* children at one birth, and all "living and doing well," may be regarded as among the extremely rare phenomena of the lying-in room. I myself have never met with an instance of such fecundity—the richest reproductive result in my practice being once, an example of triplets, two of the infants, with the mother, surviving; the third was still-born. It has been my fortune to bring thirty-three twins into the world, one of which cases I will briefly mention, as illustrating the fact that the procreation of twins seems peculiar to certain individuals and families. Some years ago I attended a lady in child-bed with twins. This lady I confined three times successively with twins. She married a German gentleman. Her husband was a twin, and his aunt on the maternal side I delivered twice consecutively of two children at each birth.

It would seem that a twin pregnancy occurs in the varying proportion of one in sixty to one in ninety-five cases. Madame La Chapelle records, that in 37,441 births that there were 36,992 single deliveries, 444 instances of twins, and but five cases of triplets; and it is an interesting fact that in 108,000 births in the Hotel Dieu, and Maternite of Paris, from the years 1761 to 1826, *there was not one example of quadruple gestation.* In 129,172 deliveries in the lying-in hospital of Dublin, there were 2,062 cases of twins; 20 of triplets; and but *one instance of a quadruple birth.* Haller (Physiologia, 929), observes, "*non raro femina geminos fœtus parit; rarius paulo tres, neque unquam supra quinque.*

Among British practitioners, in 257,935 births, according to Dr. Churchill, there were 343 cases of twins, or about 1 in 75, and 43 cases of tripulets, or 1 in 5,561½; among the French, in 38,400, there were 336 cases of twins, or 1 in 108, and 67 triplets, or 1 in 6,568; among the Germans, in 369,-080, there were 4,239 cases of twins, or 1 in 87, and 38 of triplets, or 1 in 9,765. Taking the whole, we have 666,424 cases, and 8,006 of twins, or 1 in 83, and 87 cases of triplets, or 1 in 7,443.

The following is presented as the rate of mortality: In 1,298 cases of twins (*i. e.* 2,596 children), 636 were lost, or about 1 in 4; and of 12 instances of triplets (*i. e.* 36 children),

11 were lost, or 1 in 3. The mortality to the mother in twin cases has been computed as 1 in 20.

The general rule is, that in plural pregnancy, each fœtus possesses its own membranes and placenta; and in this particular, it stimulates a single gestation, with the exception that sometimes there will be an inosculation of blood-vessels between the different placentæ. On the other hand it will occasionally, though rarely, happen that there is but one after-birth for the two children; and it has been suggested by Dr. Tyler Smith, that, in these latter instances, the one ovule has contained two yelks, and two germinal vesicles, as is sometimes observed in the case of birds—one egg with a double yelk producing two individuals. The fœtuses in plural pregnancy are usually smaller than when there is one child *in utero*, and there is, also, a strong predisposition to premature delivery. When there are more than two, the expulsion is still more apt to be premature, and the children rarely survive beyond a short time. It must, however, be admitted that there are well authenticated exceptional examples of the reverse of this latter rule. Dr. Collins cites, within his own knowledge, two instances of *triplets* having arrived at the full period of utero-gestation, and were reared healthy children.

In the great majority of cases in twin-births, statistics show that the second child is delivered, by the resources of nature alone, from fifteen to thirty minutes after the birth of the first. In 212 instances recorded by Dr. Collins, in which the interval is accurately marked, in 38, it was five minutes; in 29, ten minutes; in 48, fifteen minutes; in 23, twenty minutes; in 30, half an hour; in 5, three-quarters of an hour; in 16, one hour: in 8, two hours; in 3, three hours; in 2, six hours; in 1, seven hours; in 1, eight hours; in 1, ten hours; in 1, twenty-four hours.

In plural pregnancy, it will occasionally happen that one fœtus is healthy, and perfectly developed, while the other bears evidences of an early arrest in its growth, and may be either living or dead. This fact is very satisfactory proof that the lives of the two children are quite independent one of the other. Again, both children may be fully developed, and alive, but one much larger than the other. Cases such as I have just mentioned, may very naturally give rise to the idea of *super-fœtation*, and have been attempted to be explained by some writers exclusively upon this hypothesis; but *super-fœtation*, in my judgment, is not at all necessary for the elucidation of the phenomena—they may exist independently of any such influence. For example: this inequality may be due either to some original defect in one placenta or funis, or in one of the fœtuses; or it may result from compression exercised in utero by one child on the other. There can be no doubt of the occasional operation of either of these influences.

A plural pregnancy does not necessarily imply that the labor will not be natural; on the contrary, nature, unless there should be some complication, such as malposition of the fœtus, will be adequate to accomplish the delivery, through her own unaided resources. The labor, however, as a general rule, will be more protracted, because the uterus, having been subjected to a greater degree of distension, loses in proportion its contractile tonicity, and therefore a longer period is needed for the achievement of the process. And, also, when there is more than one fœtus in utero, the organ can not concentrate its power as in a single gestation.

The following table, exhibiting presentations of the fœtus in 808 labors with twin children, has been constructed by Prof. Simpson from the returns of twin births, as observed in the Dublin and Edinburgh Lying-in Hospitals, and among the patients of the London Maternal Charity:

| Reporter. | Total number of Cases. | Number of Head Presentations. | No. of Pelvic Presentation. | No. of Transverse Presenta'ns |
|---|---|---|---|---|
| Clarke | 126 | 73 | 53 | — |
| Collins | 449 | 309 | 133 | 7 |
| Hardy & McClintock | 190 | 122 | 62 | 6 |
| Ramsbotham | 772 | 532 | 221 | 19 |
| Simpson | 30 | 23 | 7 | — |
| Reid | 48 | 25 | 22 | 1 |
| Total | 1,615 | 1,084 | 498 | 33 |
| Proportions among twin children | | 67 in 100 | 1 in 3 | 1 in 49 |
| Proportions among all births | | 96 in 100 | 1 in 81 | 1 in 224 |

And now, my dear Doctor, I must close this letter, trusting that it will not be altogether without interest to you. You certainly have accomplished something to be proud of; and I am much pleased that you have added to your little State of South Carolina *four males*.

Believe me to be, most truly yours,

Gunning S. Bedford, M. D.

—*Correspondence of New York Medical Record.*

